# Responsiveness and construct validity of two outcome measures of bilateral upper limb function in patients with chronic stroke

**DOI:** 10.3389/fneur.2024.1352365

**Published:** 2024-05-23

**Authors:** Han-ting Tsai, Hiu-ying Lau, Keh-chung Lin, Yi-chun Li, Chia-jung Lin, Grace Yao, Ya-yun Lee, Wen-shiang Chen, Chia-ling Chen, Ya-ju Chang, Yi-shiung Horng

**Affiliations:** ^1^Department of Physical Medicine and Rehabilitation, Wan Fang Hospital, Taipei Medical University, Taipei, Taiwan; ^2^School of Occupational Therapy, College of Medicine, National Taiwan University, Taipei, Taiwan; ^3^Division of Occupational Therapy, Department of Physical Medicine and Rehabilitation, National Taiwan University Hospital, Taipei, Taiwan; ^4^Department of Occupational Therapy, College of Medicine, I-Shou University, Kaohsiung, Taiwan; ^5^Department of Psychology, National Taiwan University, Taipei, Taiwan; ^6^School and Graduate Institute of Physical Therapy, College of Medicine, National Taiwan University, Taipei, Taiwan; ^7^Department of Physical Medicine and Rehabilitation, National Taiwan University Hospital and National Taiwan University College of Medicine, Taipei, Taiwan; ^8^Department of Physical Medicine and Rehabilitation, Chang Gung Memorial Hospital at Linkou, Taoyuan, Taiwan; ^9^Graduate Institute of Early Intervention, College of Medicine, Chang Gung University, Taoyuan, Taiwan; ^10^Neuroscience Research Center, Chang Gung Memorial Hospital Linkou, Taoyuan, Taiwan; ^11^School of Physical Therapy and Graduate Institute of Rehabilitation Science, College of Medicine, Chang Gung University, Taoyuan, Taiwan; ^12^Healthy Aging Research Center, Chang Gung University, Taoyuan, Taiwan; ^13^School of Medicine, Tzu Chi University, Hualien, Taiwan; ^14^Department of Physical Medicine and Rehabilitation, Taipei Tzuchi Hospital, Buddhist Tzuchi Medical Foundation, New Taipei City, Taiwan

**Keywords:** cerebrovascular accident, rehabilitation, outcome, upper extremity, psychometrics

## Abstract

**Background:**

Stroke is a leading cause of long-term disability among stroke survivors. Despite the availability of numerous stroke rehabilitative therapies, such as mirror therapy, bilateral arm training, and robot-assisted therapy, the recovery of motor function after stroke remains incomplete. Bilateral arm function is a key component in stroke patients to perform activities of daily living and to reflect their functional autonomy.

**Objective:**

This clinimetric study investigated and compared the construct validity and responsiveness of 2 bimanual activity outcome measures, the Chedoke Arm and Hand Activity Inventory (CAHAI) and the ABILHAND Questionnaire, in individuals receiving stroke rehabilitation.

**Methods:**

The present study is a secondary analysis following the framework of the COnsensus-based Standards for the selection of health Measurement INstruments (COSMIN). Individuals with chronic stroke (*N* = 113) were recruited from outpatient rehabilitation settings. Participants received 18 to 20 sessions of robot-assisted therapy, mirror therapy, combined therapy, or conventional rehabilitation for 4 to 6 weeks. The CAHAI, ABILHAND Questionnaire, and a comparison instrument, the Motor Activity Log (MAL), were administered twice at a 4- to 6-week interval to all participants. ABILHAND scores, in logits, were converted from raw ordinal scores into a linear measure.

**Results:**

There was medium to large correlation of the CAHAI and the MAL (*ρ* = 0.60–0.62, *p* < 0.01) as well as the ABILHAND Questionnaire and the MAL (*ρ* = 0.44–0.51, *p* < 0.01). Change scores from the initial measurement to the post-intervention measurement demonstrated small to medium correlation of the CAHAI and the MAL (*ρ* = 0.27–0.31, *p* < 0.01) and medium to large correlation of the ABILHAND Questionnaire and the MAL (*ρ* = 0.37–0.41, *p* < 0.01). Overall, 7 of 8 hypotheses were supported. The hypothesis testing regarding the construct validity and responsiveness of the CAHAI and ABILHAND Questionnaire was confirmed.

**Conclusion:**

The CAHAI and ABILHAND Questionnaire are both responsive and suitable to detect changes in bilateral arm functional daily activities in individuals with chronic stroke. Patient-reported outcome measures are recommended to use along with therapist-rated outcome measures for upper limb capacity evaluation in stroke rehabilitation. Further study with a prospective study design to capture specific clinical features of participants and the use of body-worn sensors, such as the arm accelerometer, is suggested.

## Introduction

Stroke is a leading cause of disability, and the costs of post-stroke care are substantial. Despite the advances of stroke rehabilitation, more than 60% of stroke survivors do not regain full recovery of motor function for the affected side of the body due to the neurologic damage after a stroke event (1). Impairment of motor function of the affected side hinders the performance of activities of daily living (ADL) tremendously (2). Because of the limitation of the paretic arm for upper limb movement, individuals with stroke often resort to compensatory strategies to perform activities. Several studies have reported the importance of involving the paretic arm in stroke rehabilitation to minimize the learned nonuse phenomenon and the need for bilateral arm practice because bilateral arm function is a fundamental element to perform daily activities (3–5).

Many ADL tasks, such as opening a jar, wringing out a washcloth, and zipping up a zipper, require bilateral arm movement (6). Assessment for the level of bilateral arm performance facilitates the formulation of intervention regimens and outcome evaluation. At present, the Chedoke Arm and Hand Activity Inventory (CAHAI) and the ABILHAND Questionnaire are 2 commonly used outcome measures for bilateral upper limb activity (6, 7). Although the CAHAI and ABILHAND Questionnaire both assess bimanual skills in functional activities, the measures differ in their content and mode of administration. CAHAI results are based on the therapist’s rating, and the ABILHAND Questionnaire is a self-reported measure, and the results may differ in responsiveness to change and reflect differential aspects of bimanual activity. Selection of suitable instruments is a key component to outcome evaluation. Outcome measures with sound psychometric properties facilitate fair comparisons and interpretation of the results of stroke rehabilitation trials.

A previous study reported the test-retest reliability, validity, and sensitivity of the CAHAI in both acute and chronic stroke survivors (6). The CAHAI showed good inter-rater reliability, with an intra-class correlation coefficient (ICC) of 0.98 [95% confidence interval (CI), 0.96–0.99]. The total score ranges from 13 to 91. The minimal detectable change score was 6.3. Convergent and discriminant cross-sectional validity were also established for the CAHAI in this study. Another study that examined the psychometric properties and cross-cultural adaptation of the CAHAI in individuals with subacute and chronic stroke showed good construct validity and high inter-rater reliability, with an ICC of 0.97 (95% CI, 0.94–0.99) (8).

To study the metric properties of the ABILHAND Questionnaire, Wang et al. (7) investigated the validity, responsiveness, and minimal clinically important difference (MCID) of the ABILHAND Questionnaire in individuals with chronic stroke. MCID represents the smallest change in a measurement that patients perceive as beneficial and guides clinicians to assess the significance of treatment outcomes (7). The predictive validity was established based on the evaluation of the ABILHAND Questionnaire with the criterion measures of the Stroke Impact Scale and the Functional Independence Measure. The correlations among the ABILHAND and the criterion measures showed little fluctuation at the pre-treatment and post-treatment assessment, indicating the relationships among these tests were relatively stable over 4 weeks of stroke rehabilitation. The ABILHAND Questionnaire has been validated and analyzed through the Rasch model, which enabled the conversion of raw data from an ordinal scale to an interval scale (ranging from −3.5 to 6 logits) (9). The MCID range of the ABILHAND Questionnaire, estimated by Wang et al. (7), was 0.26 to 0.35. In another study, Ekstrand et al. (10) examined the test-retest reliability of the ABILHAND Questionnaire in chronic stroke survivors, which was high in the entire cohort (ICC = 0.85) and in the analysis without 4 outliers (ICC = 0.91).

Findings of the aforementioned studies suggest that the CAHAI and ABILHAND Questionnaire are both valid instruments to assess upper limb activity in individuals with stroke (7, 8, 10). Although both instruments measure bilateral upper limb activity, the CAHAI is rated by the therapist, whereas the ABILHAND Questionnaire is a patient-reported outcome measure (PROM). Recent literature has indicated the need to include PROMs in the repertoire of outcome measures in clinical rehabilitation (11). The information where the 2 instruments differ in validity and responsiveness may inform clinical practice in instrument selection. The COnsensus-based Standards for the selection of health Measurement INstruments (COSMIN) is an internationally recognized framework to evaluate measurement properties of existing PROMs (12). The COSMIN framework aims to improve the selection of measurement instruments. With regard to responsiveness, given the similar evaluation domains of bilateral upper limb performance at the ICF activity level, it was hypothesized that the CAHAI and ABILHAND Questionnaire would demonstrate comparable ability to detect clinical changes as reflected by the change score of assessments between the initial and post-intervention measurement. Based on the assumption that the more similar constructs between the outcome measure and the comparison instrument, the stronger the strength of the correlation, it was also hypothesized that the correlation of the CAHAI and the MAL was strong as both instruments evaluated the upper limb activity and were involved in assessing the movement accuracy and speed. The correlation of the ABILHAND Questionnaire and the MAL was expected to be medium. The present study compared 2 instruments through the COSMIN framework to evaluate the construct validity and responsiveness of the CAHAI and ABILHAND Questionnaire in individuals receiving stroke rehabilitation at the chronic stage.

## Materials and methods

### Participants

**Figure 1:**
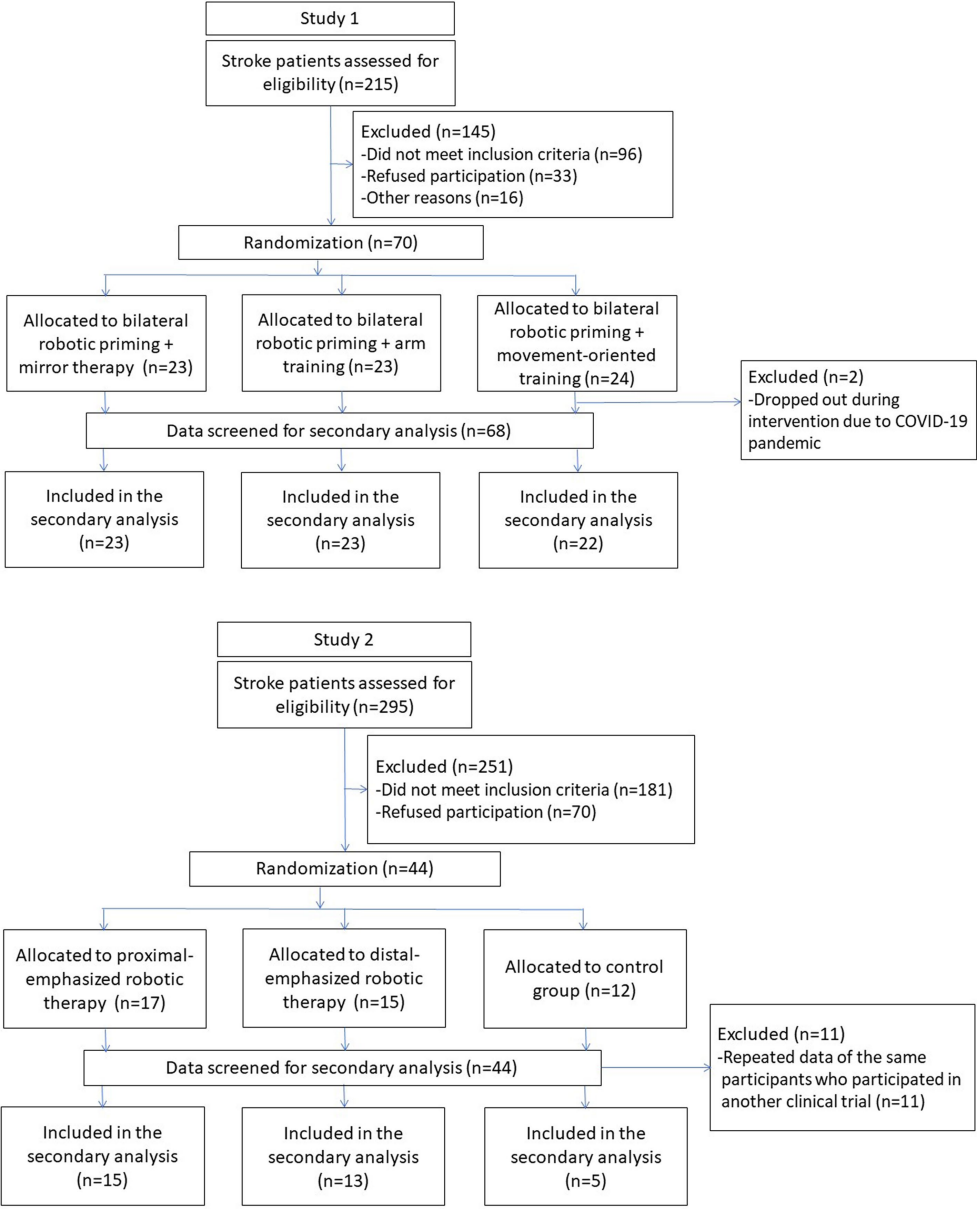

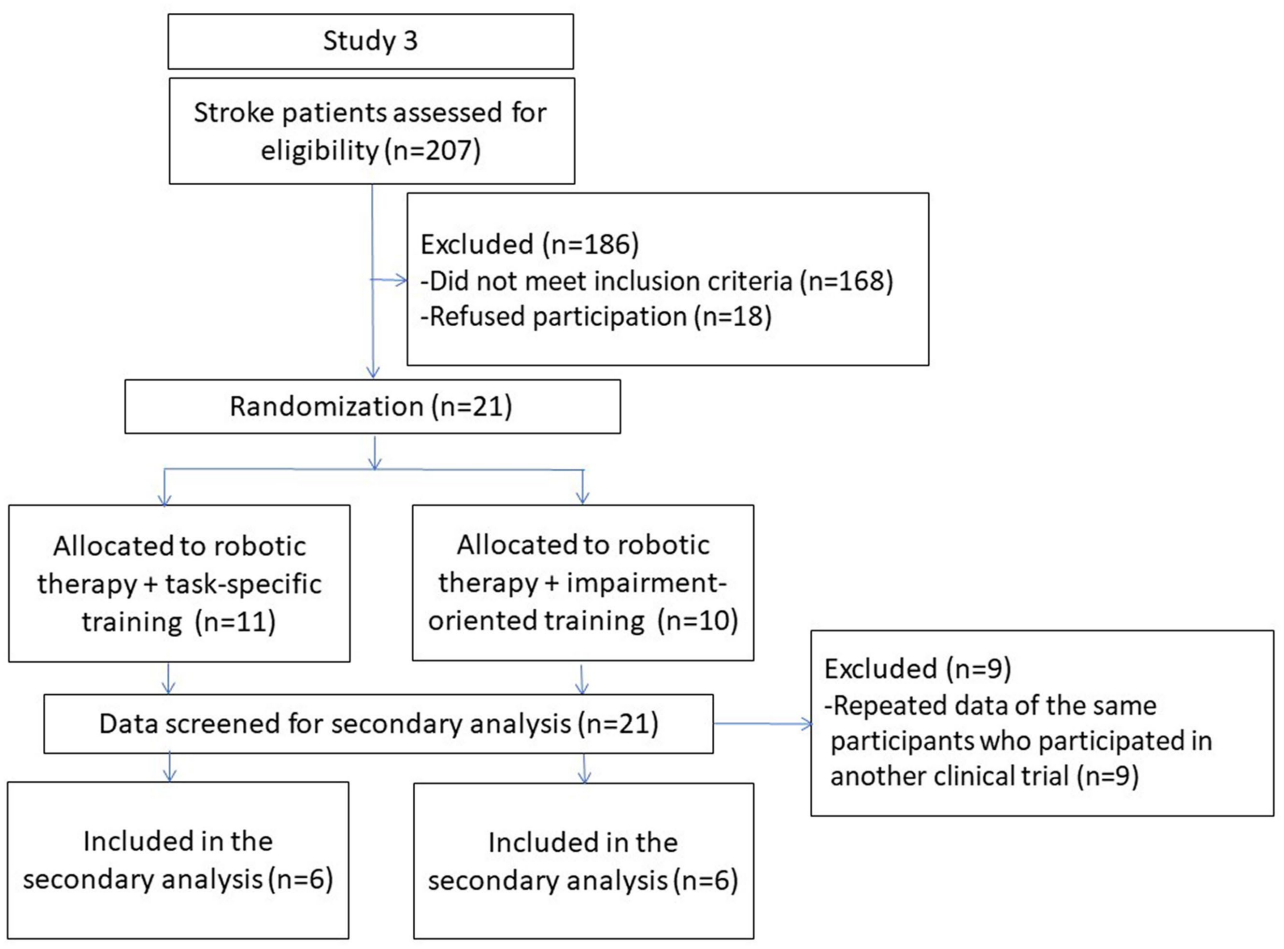
Flowcharts of study participants recruited to the secondary analysis from 3 previous clinical trials.

This study was a secondary analysis of data from 113 participants collected from 3 randomized clinical trials of stroke motor rehabilitation of the upper limb (13–15) (Figure 1). If the participants joined more than one clinical trial, only the first set of data was included in the current analysis to minimize any contamination of study data. Participants with the following criteria were eligible for inclusion: (1) a first-ever unilateral stroke confirmed by brain imaging and diagnosed by a clinician according to International Classification of Diseases, 10th Revision code I60–I63, (2) at least 3 months after stroke onset, (3) between 20 and 80 years old, and (4) an initial score of >10 in Fugl-Meyer Assessment for Upper Extremity subscale (FMA-UE). The exclusion criteria were (1) comorbidity with major neurologic, neuropsychologic, or orthopedic diseases, (2) Modified Ashworth Scale >3 in any joints of the paretic upper limb, (3) severe visual impairment, and (4) participation in other clinical trials during the study period. The Institutional Review Board of the participating sites reviewed and approved the studies. All participants signed the written informed consent before the studies began.

### Intervention and procedures

The participants went through robot-assisted therapy (RT), mirror therapy (MT), hybrid therapy, or conventional rehabilitation in the outpatient occupational therapy units of the study sites. RT involved passive and active training modules of forearm pronation/supination and wrist flexion/extension. Participants performed approximately 1,200 to 1,600 repetitions in 40 to 45 min.

MT was performed with a wooden mirror box, measuring 41 × 50 × 30 cm^3^, that was positioned on the table in front of where participants were seated. The participants were instructed to focus on the mirror reflection of the less affected arm during the training. The MT consisted of a range of motor movements, including gross motor movement, fine motor movement, and object manipulation. Specifically, the tasks involved picking up a piece of snack from a bag, transferring pegs on a pegboard, or other functional tasks.

The conventional rehabilitation included passive or active range of motion exercise, muscle strengthening, and ADL task practice.

The treatment dosage and training frequency of participants from the 3 clinical trials were comparable. All participants received 18 to 20 sessions of treatment in 4 to 6 weeks. The frequency of training was 3 to 5 days per week. Each treatment session lasted 90 to 100 min. Certified occupational therapists conducted the interventions. The initial and final outcome measures were administered by the same person. The raters were 1 experienced therapist and 2 well-trained research assistants, who were blind to the group allocation and were not involved in any screening or treatment procedures of the study. All assessments were conducted according to the sequence of the study protocol of the respective clinical trials.

### Measures

Hypotheses for the present study were formulated before the analyses with the data of the available measures used for the randomized controlled trial. Only the study data relevant to the hypotheses for the present study were used. Two outcome measures of bilateral upper limb activity, the CAHAI and the ABILHAND Questionnaire, were included in the present study. Also included was the Motor Activity Log (MAL), which assessed the quality and amount of movement during daily functional tasks. These 3 instruments encapsulate the definition of “Activity” in The International Classification of Functioning, Disability and Health framework (16), which is the execution of a task or action by an individual, reflecting their capacity despite any impairments.

#### Chedoke Arm and Hand Activity Inventory

The CAHAI was developed on the basis of involving bilateral upper limbs to perform functional tasks and has established reliability and validity (6). The CAHAI is a performance-based outcome measure that consists of 13 items related to real-life activities (6, 8). Each item is assessed according to the standardized protocol and rated by a therapist. Responses to the inventory are recorded using a 7-point quantitative scale ranging from “total assistance” (1 point) to “independent” (7 points). A higher CAHAI score represents better performance in functional tasks.

#### ABILHAND Questionnaire

The ABILHAND Questionnaire was developed with the key feature of measuring the individual’s self-perceived difficulty in performing daily activities that involve the use of the bilateral upper limbs (7). The questionnaire is a self-reported outcome measure that comprises 23 items of everyday life activities. The respondent assesses and rates each item on a 4-point scale that covers “impossible” (0 points), “very difficult” (1 point), “difficult” (2 points), and “easy” (3 points). The ABILHAND raw data collected from the study were first converted to a Rasch logits score before data analysis was performed.^1^ The estimated Rasch-based logits of the ABILHAND Questionnaire in chronic stroke patients, validated by Penta et al. (9), is between −3.5 and 6 logits. A higher logit value indicates a higher ability to perform functional tasks that require movements of the bilateral upper limbs.

#### Motor Activity Log

The MAL is a self-perceived assessment tool for hemiparetic stroke patients to measure the spontaneous use of their paretic arm and hand [amount of use (AOU) and quality of movement (QOM)] in daily activities (17). The MAL is administered by semistructured interviews in which patients are asked to rate how much and how well they were able to use their paretic arm and hand to accomplish each of the 30 ADLs listed in the MAL. The aggregate scores range from 0 to 150 for the domains of AOU and QOM. AOU scores range from 0 (never use the affected arm for this activity) to 5 (always use the affected arm for this activity), and QOM scores range from 0 (inability to use the affected arm for this activity) to 5 (ability to use the affected arm for this activity just as well as before the stroke).

### Data analysis

The COSMIN checklist provides criteria to evaluate construct validity and responsiveness (18). Analyses were based on the COSMIN recommendations. The present study compared the CAHAI and ABILHAND Questionnaire with another outcome measurement instrument, the MAL; therefore, the hypothesis testing for construct validity and responsiveness was used.

#### Construct validity

Construct validity describes the degree to which the scores of health-related outcome instruments are consistent with a hypothesis formulated before data analysis, based on the assumption that the instrument validly measures the construct of interest (19). According to the COSMIN recommendation, the hypotheses were formulated for expected correlations between the CAHAI and the MAL and the expected correlations between the ABILHAND Questionnaire and the MAL. The CAHAI and the MAL were expected to have strong correlation based on 2 similarities of the assessed constructs, which include arm and hand involvement in performing ADL tasks and performance speed (20). The ABILHAND Questionnaire and the MAL were expected to have at least a medium correlation. Four hypotheses regarding the construct validity were tested:

*H1*: The CAHAI will have a positive correlation of at least 0.5 with the MAL-AOU.

*H2*: The CAHAI will have a positive correlation of at least 0.5 with the MAL-QOM.

*H3*: The ABILHAND will have a positive correlation of at least 0.3 with the MAL-AOU.

*H4*: The ABILHAND will have a positive correlation of at least 0.3 with the MAL-QOM.

#### Responsiveness

Responsiveness refers to the degree to which an instrument is able to measure change in the construct to be measured (21). To examine the responsiveness, hypotheses regarding the relationship between the change scores of the CAHAI and the MAL and the change scores of the ABILHAND Questionnaire and the MAL between the initial measurement and the post-intervention measurement were formulated. Change scores for all measurements were calculated by subtracting the post-intervention scores from the initial measurement scores. All 3 outcome measures—the CAHAI, ABILHAND Questionnaire, and the MAL—belong to the activity domain of the International Classification of Functioning, Disability and Health framework (20). The correlation of change scores between the CAHAI and the MAL and between the ABILHAND Questionnaire and the MAL were expected to have medium to large effect based on the assumption that all 3 outcome measures would reflect changes in overall activity execution. Another 4 hypotheses regarding the responsiveness were tested:

*H5*: A positive correlation of 0.3 to 0.49 is expected between the change score of the CAHAI and the change score of the MAL-AOU.

*H6*: A positive correlation of 0.3 to 0.49 is expected between the change score of the CAHAI and the change score of the MAL-QOM.

*H7*: A positive correlation of 0.3 to 0.49 is expected between the change score of the ABILHAND and the change score of the MAL-AOU.

*H8*: A positive correlation of 0.3 to 0.49 is expected between the change score of the ABILHAND and the change score of the MAL-QOM.

#### Data analysis

The analyses were conducted using IBM SPSS Statistics 26.0. Missing data would be excluded from the analyses using the pairwise method. According to the COSMIN checklist, a sample size for testing measurement properties of *n* ≥ 100 is very good; *n* = 50 to 99 is adequate; *n* = 30 to 49 is doubtful, and *n* < 30 is inadequate (18). The Spearman rank correlation coefficient (*ρ*) was computed for ordinal scales. The Cohen definition was used to indicate the strength of the correlation indicated by the classification of small (0.10 to 0.29), medium (0.30 to 0.49), and large (0.50 to 1.0) (22). The level of significance for all analyses was set at *p* < 0.05.

## Results

The analysis included the data for 113 participants (79 men and 34 women) who completed the assessments and stroke rehabilitation, which was a sufficient sample size for evaluating the construct validity and responsiveness of the CAHAI and ABILHAND Questionnaire. There were no missing data during the process of data analysis. The participants were a mean age of 54.77 years. Table 1 summarizes the demographic background and baseline clinical characteristics of the study participants.

**Table 1:** Demographic and baseline clinical characteristics of the participants (*N* = 113).

Characteristics	Data value
*Sex*	
Male	79
Female	34
Age, (y)	54.77 ± 12.38
*Side of stroke*	
Right	67
Left	46
Months after stroke	19.27 ± 14.00
CAHAI	38.14 ± 14.74
ABILHAND at pretest, logits score	−0.33 ± 0.97
MAL-AOU	27.73 ± 21.00
MAL-QOM	22.50 ± 19.15

Data are presented as the number of patients (*N* = 113) or as the mean ± standard deviation. AOU, amount of use; CAHAI, Chedoke Arm and Hand Activity Inventory; MAL, Motor Activity Log; QOM, quality of movement.

Table 2 reports the correlations between the outcome measures at the initial measurement point. The results in relation to the construct validity hypotheses were as follows:

*H1*: The correlation between the CAHAI and the MAL-AOU was 0.60 (*p* < 0.01), and a large positive correlation supported hypothesis 1.

*H2*: The correlation between the CAHAI and the MAL-QOM was 0.62 (*p* < 0.01), and a large positive correlation supported hypothesis 2.

*H3*: The correlation between the ABILHAND Questionnaire and the MAL-AOU was 0.44 (*p* < 0.01), and a medium to large positive correlation supported hypothesis 3.

*H4*: The correlation between the ABILHAND Questionnaire and the MAL-QOM was 0.51 (*p* < 0.01), and a large positive correlation supported hypothesis 4.

**Table 2:** Correlations between assessments at the initial measurement point (*N* = 113).

Correlations between	Expected	Observed
CAHAI and MAL-AOU	≥0.5	**0.60** ^*^
CAHAI and MAL-QOM	≥0.5	**0.62** ^*^
ABILHAND and MAL-AOU	≥0.3	**0.44** ^*^
ABILHAND and MAL-QOM	≥0.3	**0.51** ^*^

AOM, amount of use; CAHAI, Chedoke Arm and Hand Activity Inventory; MAL, Motor Activity Log; QOM, quality of movement. Correlations: Spearman’s *ρ*, ^*^p < 0.01. The bold values indicate the observed value of correlation coefficient is in accordance with the expected value.

All of the hypotheses for construct validity testing were supported and confirmed. In addition, all of the 4 hypothesis testing results (hypothesis 1 to hypothesis 4) were statistically significant.

Table 3 provides the correlations of the change score of assessments between the initial and post-intervention measurement point. The results in relation to the responsiveness hypotheses were as follows:

*H5*: The correlation between the change score of the CAHAI and the change score of the MAL-AOU was 0.27 (*p* < 0.01), but the magnitude of the observed value was slightly lower than the expected value.

*H6*: The correlation between the change score of the CAHAI and the change score of MAL-QOM was 0.31 (*p* < 0.01), and a medium to large positive correlation supported hypothesis 6.

*H7*: The correlation between the change score of ABILHAND Questionnaire and the change score of the MAL-AOU was 0.41 (*p* < 0.01), and a medium to large positive correlation supported hypothesis 7.

*H8*: The correlation between the change score of the ABILHAND Questionnaire and the change score of the MAL-QOM was 0.37 (*p* < 0.01), and a medium to large positive correlation supported hypothesis 8.

**Table 3:** Correlations of the change score of assessments between the initial and post-intervention measurement (*N* = 113).

Correlations between	Expected	Observed
Change score of CAHAI and change score of MAL-AOU	0.3–0.49	0.27^*^
Change score of CAHAI and change score of MAL-QOM	0.3–0.49	**0.31** ^*^
Change score of ABILHAND and change score of MAL-AOU	0.3–0.49	**0.41** ^*^
Change score of ABILHAND and change score of MAL-QOM	0.3–0.49	**0.37** ^*^

AOM, amount of use; CAHAI, Chedoke Arm and Hand Activity Inventory; MAL, Motor Activity Log; QOM, quality of movement. Correlations: Spearman’s *ρ*, ^*^p < 0.01. The bold values indicate the observed value of correlation coefficient is in accordance with the expected value.

Three of four hypotheses achieved the expected magnitude of correlation values. In terms of magnitude of correlations, hypotheses 6, 7, and 8 were supported and confirmed. The 4 hypothesis tested (hypothesis 5 to hypothesis 8) were statistically significant.

In sum, for the CAHAI, 2 of 2 hypotheses for construct validity and 1 of 2 for responsiveness were supported; for the ABILHAND Questionnaire, 2 of 2 hypotheses for construct validity and 2 of 2 for responsiveness were supported.

## Discussion

As far as we know, this is the first retrospective study to investigate the construct validity and responsiveness of the CAHAI and ABILHAND Questionnaire for individuals with chronic stroke using the COSMIN framework. Overall, 7 of 8 research hypotheses were supported, which fulfilled the COSMIN guideline with the rule of thumb to obtain at least 75% of results being in accordance with the *a priori* hypotheses so that the study result was sufficient to accept the validity and responsiveness (18). The research hypotheses of the current study regarding the responsiveness of the CAHAI and ABILHAND Questionnaire, and the strength of correlation of these two outcome measures with the MAL, were supported by the overall results. The findings of the present study may be generalized to individuals with a first-ever unilateral stroke for at least 3 months after stroke onset. However, stroke survivors with different impairment levels and time of onset may require further study to confirm whether the findings are applicable.

As hypothesized, at the initial measurement point, the correlations of the CAHAI and MAL-AOU (0.60) and the CAHAI and MAL-QOM (0.62) were high, as expected, which may provide further evidence to the construct validity of the CAHAI to the validation study by Barecca et al. (6) and Choo et al. (8). The results supported the hypothesis of overlapping constructs of the CAHAI and the MAL that both evaluate the performance of activity involving the use of arms, speed, and precision. The results of at least medium correlations of the ABILHAND Questionnaire and MAL-AOU (0.44) and the ABILHAND Questionnaire and MAL-QOM (0.51) were in accordance with the hypotheses. In fact, the ABILHAND Questionnaire and MAL-QOM even attained a strong positive correlation. One possible reason for the strong correlation of the ABILHAND Questionnaire and MAL-QOM may be due to the same rating method that both are PROMs and call on the stroke individual’s own perception of activity capability and performance.

The CAHAI and ABILHAND Questionnaire both appeared responsive. The results of the present study align with the study of Barreca et al. (6) and Wang et al. (7) that the CAHAI and ABILHAND Questionnaire are sensitive to detect change for individuals receiving stroke rehabilitation. The scores of the ABILHAND Questionnaire from the post-intervention measurement changed in the expected magnitude and direction indicated by the comparison of another instrument, the MAL. The magnitude of correlation between the change in the CAHAI and the change in MAL-AOU (0.27) was slightly lower than the expected value (0.30–0.49) from the *a priori* hypothesis. Although the strength of correlation between the change in the CAHAI and change in the MAL-AOU was small, the correlation was statistically significant. The low correlation may be because the MAL-AOU primarily assesses how much the stroke survivors use their more affected arm to perform daily activities in real life. However, individuals with stroke may seek help from family members or they use compensation strategies to complete certain tasks in real-life situations. On the other hand, the MAL-QOM captures the speed, accuracy, and the amount of effort required for movement in the more affected arm. The CAHAI is also involved in the evaluation of movement, speed, and precision. This may be a possible explanation of why the CAHAI and the MAL-QOM attained the expected magnitude of correlation in hypothesis 6.

Several studies have reached consensus that the Action Research Arm Test (ARAT) is one of the core sets of motor outcome measures in stroke rehabilitation (23, 24). The ARAT has been used to evaluate the upper limb activity limitations of patients with stroke, multiple sclerosis, and traumatic brain injury (20). The aggregated evidence contributes to the strong psychometric properties of the ARAT.

The CAHAI and ABILHAND Questionnaire have gained popularity recently for in-clinic assessment and clinical studies because both of them involve assessing real-life functional tasks that reflect a patient’s activity performance in daily life. Besides, 3 short versions of the CAHAI are available with 7, 8, and 9 tasks (CAHAI-7, CAHAI-8, and CAHAI-9) for clinical assessment (8). The short versions of the CAHAI take a shorter time to administer, and their psychometric properties are comparable to the original version.

The ABILHAND Questionnaire is also a cost-effective PROM. A recent study by Prange-Lasonder et al. (11) indicated that the CAHAI and ABILHAND Questionnaire both have strong evidence and psychometric properties in stroke rehabilitation, as evaluated by systematic reviews and expert consensus. The reviews and expert consensus recommended that PROM should be used along with a therapist-rated outcome measure. The findings of the present study suggest that both the CAHAI and ABILHAND Questionnaire are suitable to measure stroke individual’s changes due to motor rehabilitation of the upper limb. This study provides empirical evidence that may inform the instrument selection of stroke outcome evaluation for both clinicians and researchers. Both the CAHAI and ABILHAND Questionnaire may supplement each other and may be used concurrently for comprehensive evaluation of bilateral upper limb activity in stroke rehabilitation.

The reviews and expert consensus also recommended the use of body-worn sensors to measure actual arm use and monitor activity performance (11). The study by Chen et al. (25) reported the arm accelerometers demonstrated acceptable predictive validity and responsiveness in chronic stroke survivors. The MCID range for the arm accelerometer was 547 to 751 mean counts. The arm accelerometers offer round-the-clock monitoring of actual arm use. The device provides more information about movement quality and difficulties encountered, such as more or less time may have been required for a certain movement and may also delineate spontaneous use of the more affected arm and compensation. Future study is recommended to include kinematic measures as an additional outcome parameter to measure the real-life arm physical activity in stroke clinical trials (26).

### Study limitations

This study has some limitations. First, this is a retrospective study that used the existing data with limited scope. Only individuals with chronic stroke with a specific age range and clinical features were included; thus, the results of this study may not be generalized to stroke survivors with other characteristics. A prospective approach may be considered for future study to include more detailed and specific data.

Second, the treatment groups in the present study received RT, MT, and conventional rehabilitative therapy. Responsiveness to the change of the measures of bilateral arm activity may be specific to the treatment and dose. The diversified treatment regimens may have contributed to the variation of treatment effects. Further research is recommended to validate the responsiveness and investigate the metric soundness of the CAHAI and ABILHAND Questionnaire based on study cohorts receiving specific interventions.

## Conclusion

The results of this study using the COSMIN guideline and checklist support the construct validity and responsiveness of the CAHAI and ABILHAND Questionnaire for individuals with chronic stroke receiving rehabilitative therapies. The CAHAI and ABILHAND Questionnaire are both responsive to changes reflected by the performance of ADL required for community life. Evidence of this study may add to the existing literature of validation research to enrich the body of evidence. The findings of the present study suggest that the evaluation for the activity of the bilateral upper limbs should include both the performance-based test such as the CAHAI and the patient self-reported measure such as ABILHAND Questionnaire to capture the diverse profiles of motor recovery after stroke. Therapist rating scale and PROM may complement each other in the assessment for motor rehabilitation outcomes. Further study may extend to validate the findings based on study cohorts receiving unilateral and bilateral arm training.

## Data availability statement

The raw data supporting the conclusions of this article will be made available by the corresponding author, without undue reservation.

## Ethics statement

The studies involving humans were approved by Institutional Review Board and Ethics Committee of National Taiwan University Hospital and all participating clinical settings. The studies were conducted in accordance with the local legislation and institutional requirements. The participants provided their written informed consent to participate in this study.

## Author contributions

H-tT: Formal analysis, Methodology, Writing – original draft, Writing – review & editing. H-yL: Formal analysis, Methodology, Writing – original draft, Writing – review & editing. K-cL: Conceptualization, Funding acquisition, Supervision, Writing – review & editing. Y-cL: Data curation, Software, Validation, Writing – review & editing. C-jL: Project administration, Writing – review & editing. GY: Writing – review & editing. Y-yL: Writing – review & editing. W-sC: Writing – review & editing. C-lC: Writing – review & editing. Y-jC: Writing – review & editing. Y-sH: Writing – review & editing.

## Footnotes

^1^Rasch software; RUMM Laboratory.
